# Icariin in Combination with Amoxycillin-Clavulanate and Ampicillin, but Not Vancomycin, Increases Antibiotic Sensitivity and Growth Inhibition against Methicillin-Resistant *Staphylococcus aureus*

**DOI:** 10.3390/antibiotics11020233

**Published:** 2022-02-11

**Authors:** María Cardells Peris, Alba Martínez, Marina Pascual Ortíz, Chirag C. Sheth, Veronica Veses

**Affiliations:** Faculty of Health Sciences, Universidad CEU Cardenal Herrera, C/Ramon y Cajal s/n, Alfara del Patriarca, 46115 Valencia, Spain; maria.cardells@alumnos.uchceu.es (M.C.P.); alba.martinezalbinana@uchceu.es (A.M.); marina.pascual@uchceu.es (M.P.O.)

**Keywords:** antibiotic resistance, *Staphylococcus aureus*, MRSA, icariin, enhancement, amoxycillin-clavulanate, ampicillin, vancomycin

## Abstract

The widespread irrational use of antibiotics in recent years has resulted in an increase in the detection of multi-resistant bacterial strains, particularly methicillin-resistant *Staphylococcus aureus* (MRSA). The use of natural derivatives such as flavonoids is postulated as one of the most promising avenues to solve this emerging public health problem. The objective of the present study is to characterize the antimicrobial activity of icariin, a flavonoid compound isolated from a variety of plants of the *Epimedium genus*, against human and animal clinical MRSA isolates. Our study found that icariin alone did not have any antimicrobial effect on *S. aureus* or MRSA clinical isolates. However, icariin enhanced the effect of amoxycillin-clavulanate or ampicillin, whereas no effect was seen when used in combination with vancomycin. Specifically, co-incubation of *S. aureus* with amoxycillin-clavulanate plus icariin resulted in an increased proportion of dead cells, suggesting that this flavonoid potentially increases antimicrobial activity when used in combination with the beta-lactam antibiotic amoxycillin-clavulanate. Furthermore, we demonstrate that co-incubation of *S. aureus* with AmoxyClav plus icariin resulted in increased membrane disruption and growth inhibition. This study demonstrates the potential utility of icariin in permitting lower antibiotic therapeutic doses in alignment with strategies to reduce the spread of antibiotic resistance. Further research is required to determine the optimum concentration of icariin and to define clinically relevant combinations of flavonoid and antibiotic.

## 1. Introduction

The widespread irrational use of antibiotics has resulted in recent years in an increase in the detection of multi-resistant bacterial strains in clinical settings all over the world. Among them, *Staphylococcus* spp. infections have become a pressing problem in clinical practice [1,2] due primarily to the elevated resistance to new and commonly prescribed antibiotics, leading to a shortfall of therapeutic arsenal to combat these infections. This is postulated to arise due to selective pressure imposed by therapeutic misuse and abuse of antibiotics [3]. Antimicrobial resistance, especially in *Staphylococcus aureus*, represents one of the biggest current public health problems [4]. In the European Union, each year, health agencies report more than 670,000 infections caused by bacteria resistant to antibiotics, in addition to approximately 33,000 deaths as a direct consequence of these kinds of infections [5].

According to Wang et al., the current lack of sufficient and effective antimicrobial therapeutic options and the scarcity of interest among pharmaceutical companies for developing novel antimicrobial drugs may lead to serious complications arising from common bacterial infections in the near future [6]. The requirement for novel solutions to the problem of rising antimicrobial resistance has encouraged research into the use of natural products such as flavonoids as therapeutic agents [7]. Clinical studies show that natural compounds such as flavonoids may be suitable candidates for antimicrobial therapy, either alone or in combination with conventional antibiotics [8,9,10,11,12,13,14,15,16,17,18,19,20,21].

**Figure 1:**
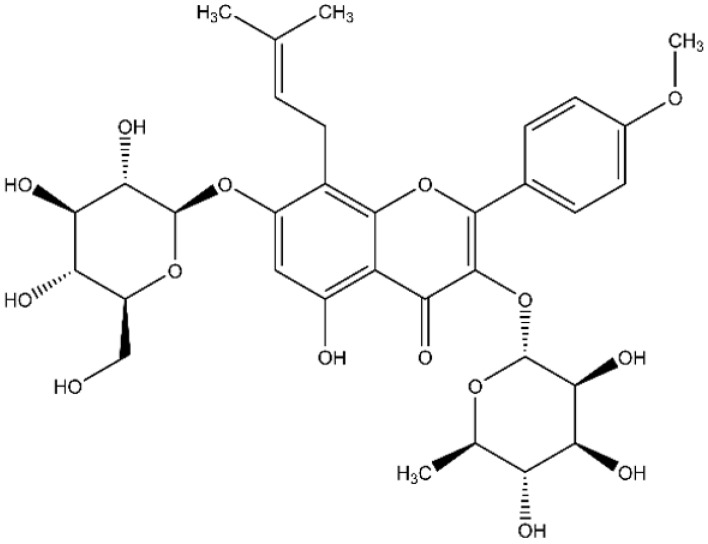
Chemical structure of icariin.

In this context, some authors have highlighted the potential antimicrobial properties of the natural flavonoid icariin [22]. This compound is isolated from the caudex and frond of a variety of plants of the *Epimedium* genus, and its chemical structure is 3-[(6-deoxy-alpha-L-mannopyranosyl)oxy]-7-(beta-D-glucopyranosyloxy)-5-hydroxy-2-(4-methoxyphenyl)-8-(3-methyl-2-buten-1-yl)-4H-1-benzopyran-4-one, as shown in Figure 1 [23]. A review of the scientific literature reveals multiple pharmacological effects of icariin in humans, including effects on angiogenesis, induction of apoptosis, neuroprotective properties, preservation of chondrocytes, and inhibition of osteoclast differentiation [24,25,26,27]. In regards to its potential antimicrobial properties, Coneye et al. described that the use of icariin at 0.08% (*w*/*v*) inhibits the growth of *Propionibacterium acnes* in vitro up to 70%, while a concentration of 0.01% inhibited the growth of *P. acnes* up to 40%, thereby suggesting a dose-dependent relationship [22]. Wang et al. published those aromatic sulfonyls in C-3 substituted icariin derivatives may represent a novel class of anti-MRSA agents (methicillin-resistant *S. aureus*; MRSA) [6]. These substances were shown to bind to the allosteric site of penicillin-binding protein 2a (PBP2a; responsible for the antimicrobial resistance of MRSA), resulting in an inhibition of cell wall synthesis [6].

The objective of the present study was to characterize the antimicrobial activity of purified, commercially sourced icariin against multiple human and animal clinical MRSA isolates, in addition to a culture collection-typed *S. aureus* strain. Icariin was used alone or in combination with antibiotics (amoxycillin-clavulanate (AmoxyClav), ampicillin and vancomycin) in order to evaluate its potential for combined use in clinical practice. As a secondary objective of the study, we undertook to broadly characterize the mode of action of icariin in inhibiting bacterial growth.

## 2. Results

### 2.1. Evaluation of the Antimicrobial Effect of Icariin on Laboratory and Clinical Isolates of Methicillin-Resistant Staphylococcus aureus

The Kirby-Bauer method was used to assess the potential antimicrobial activity of icariin against different strains of *S. aureus*, as it is a robust and established method in clinical practice [28]. All strains tested are described in Table 1, including one culture collection type strain (control) and several human and animal clinical isolates of MRSA. No antimicrobial effect was seen when the *S. aureus* isolates were grown in media supplemented only with icariin (1% *w*/*v*) in the absence of antibiotics (Supplementary Table S1).

**Figure 2:**
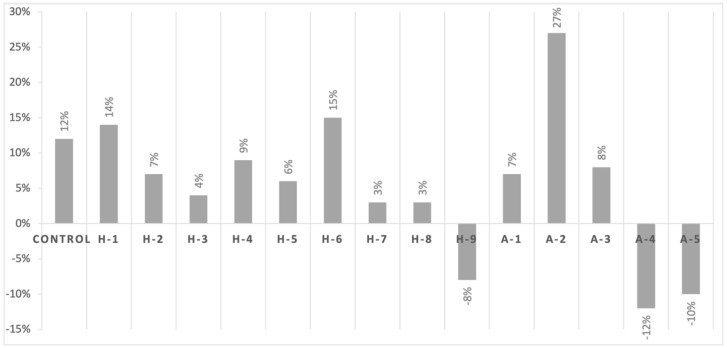
Percentage increase or decrease in the diameter of the inhibition zone when icariin was used in combination with AmoxyClav. All values are expressed in comparison with the control strain. Complete descriptions of the abbreviated strain names on the *x*-axis can be found in Table 1.

The control strain showed an increase in sensitivity of 12% when incubated with AmoxyClav in combination with icariin 1% (*w*/*v*). Most MRSA human clinical isolates, as well as animal clinical isolates of *mecA*+, showed an increase of 3% to 27% in sensitivity to AmoxyClav, while a decrease in sensitivity between −8% and −12% was observed in one human MRSA isolate (H9) and *mecC*+ animal clinical isolates (Figure 2).

**Figure 3:**
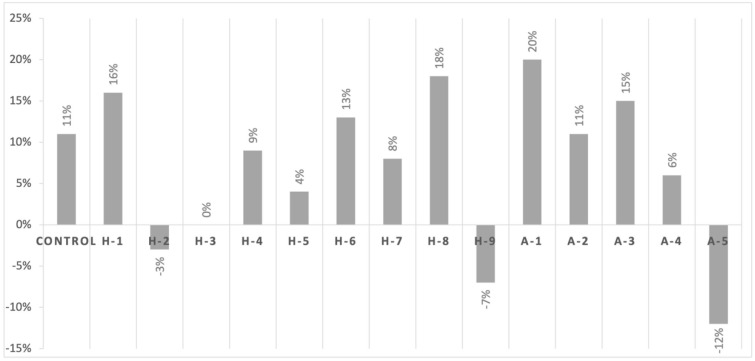
Percentage increase or decrease in the diameter of the inhibition zone when icariin is used in combination with ampicillin in comparison with the control strain. Complete descriptions of the abbreviated strain names on the *x*-axis can be found in Table 1.

When a combination of icariin (1% *w*/*v*) plus ampicillin was used, the control strain showed an increase of 11% in the diameter of the inhibition zone. Furthermore, we observed an increase in sensitivity in all *mecA*+ animal clinical isolates (increase in diameter of the inhibition zone of between 11% and 20% with respect to the control). The MRSA human clinical isolates showed a general trend of increased susceptibility with respect to the control one, while the MRSA *mecC*+ animal clinical isolates showed a range between −12% and 6% (Figure 3).

**Figure 4:**
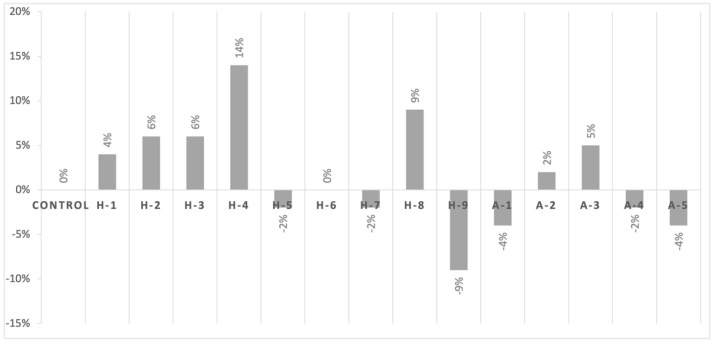
Percentage increase or decrease in the diameter of the inhibition zone when icariin is used in combination with vancomycin in respect to the control strain. Complete descriptions of the abbreviated strain names on the *x*-axis can be found in Table 1.

When vancomycin in combination with icariin 1% (*w*/*v*) was used, we did not observe any difference in the control strain. Similarly, all MRSA *mecA*+ animal isolates showed no significant differences in the diameter of the inhibition zones. On the other hand, the clinical isolates *mecC*+ showed a decreased sensitivity to vancomycin when icariin was added (Figure 4). The MRSA human clinical isolates displayed a trend toward increased susceptibility, although two strains showed a slight decrease in susceptibility (H5 and H7), and one of them (H6) showed no difference in behavior when icariin was used in combination with vancomycin.

### 2.2. Assessment of Cell Viability in the Presence of Antibiotics with Icariin

**Figure 5:**
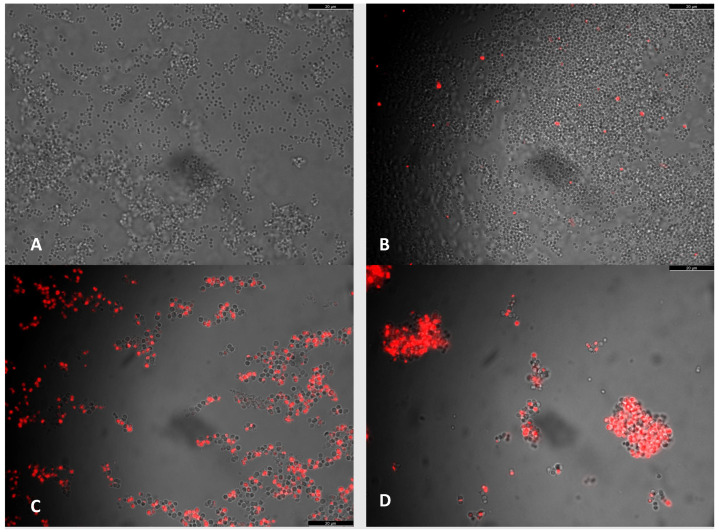
Phase-contrast and fluorescent microscopy of *S. aureus* CECT435. (**A**) Control strain grown in BHI liquid at 3 h. (**B**) Control strain incubated in brain heart infusion (BHI) liquid media supplemented with icariin to a final concentration of 30 µg/mL at 3 h. (**C**) Control strain incubated in BHI liquid media supplemented with AmoxyClav at 3 h. (**D**) Control strain incubated in BHI liquid media supplemented with icariin + AmoxyClav.

To study the effect of co-incubation of icariin and AmoxyClav, the cell viability of *S. aureus* control strain was assessed in liquid cultures grown with icariin, antibiotic, and both, using the propidium iodide vital staining (Figure 5). This experiment demonstrated that co-incubation of *S. aureus* with AmoxyClav plus icariin resulted in an increased proportion of dead cells (Figure 5D). This effect was deemed to be enhanced in the presence of icariin as the proportion of dead cells in co-incubated cultures was greater than in cultures where the antibiotic alone was used (Figure 5C).

To analyze the effect of icariin when administered along with AmoxyClav on *S. aureus* growth inhibition, colony-forming units (CFU) counts were performed following co-incubation of the control strain with icariin, AmoxyClav, or a combination of both (Table 2). Exposure of the control strain to icariin in combination with AmoxyClav resulted in a growth inhibition, as demonstrated by a reduction in CFU from 1,18E + 06 (*BHI* + *AmoxyClav*; Table 2) to 8,61E + 05 (*BHI* + *AmoxyClav* + *icariin*; Table 2). A student’s t-test determined that this difference was statistically significant (*p* = 0.01). These results represent a statistically and clinically significant reduction in the number of CFU of 37,4% at three hours with respect to the samples treated with antibiotics alone. Additionally, we demonstrate that the addition of icariin on its own (*BHI* + *icariin*; Table 2) did not result in a statistically significant difference in CFUs with respect to the control (*BHI*; Table 2), thereby supporting our hypothesis suggesting that icariin acts in combination with antibiotics to enhance the bacterial susceptibility of *S. aureus*.

## 3. Discussion

During the last decade, numerous research papers have been published regarding the combination of conventional antibiotics with natural extracts from plants as a new strategy against infections caused by multi-resistant bacteria. The overall objectives of these articles include searching for molecules that act to enhance the effect of conventional antibiotics. Such molecules could potentially reduce the antibiotic dose to the patient, reduce treatment duration or enhance the killing effect, and as such, avoid contributing to increasing antibiotic resistance in clinical bacterial isolates. Several flavonoids have been described to have synergistic activity against multi-resistant strains of *S. aureus*. However, there are no known studies on the synergistic properties of icariin, a flavonoid extracted from a variety of plants of the *Epimedium* genus [23], alone or in combination with conventional antibiotics in clinical practice.

Our study found that the combination of icariin 1% (*w*/*v*) with AmoxyClav increased the effectiveness of the activity of the antibiotic by approximately 12% against human and animal clinical MRSA isolates. Animal isolates characterized as being *mecA*+ or *mecC*+ showed a significant difference in response to icariin plus antibiotic co-incubation. Antibiotic sensitivity increased by up to 27% in *mecA*+ isolates while we observed a decrease in sensitivity of up to 12% in *mecC*+ strains, suggesting that these genotypic differences may play a role in determining the effectiveness of icariin-specific enhancement in the treatment of MRSA clinical isolates.

When icariin was co-incubated with ampicillin, this resulted in an increase in sensitivity to the antibiotic of 11% in culture collection type strain. Sensitivity to ampicillin increased by up to 20% in *mecA*+ animal clinical isolates, while we observed a decrease in sensitivity of up to 12% in *mecC*+ animal clinical isolates.

MRSA strains produce an altered PBP2a associated with decreased affinity for most semisynthetic penicillins. The protein is encoded by an acquired gene, *mecA*, and its new homologs (*mecB*, *mecC*, and *mecD*) carried on a mobile genetic element (MGE) designated staphylococcal cassette chromosome *mec* (SCC*mec*) [29]. In this context, *mecC*-carrying MRSA has been described as strains presenting stronger virulence factors and higher levels of antimicrobial resistance [30]. We hypothesize that this could explain the different behavior observed between the *mecA*+ and *mecC*+ animal clinical isolates.

Lastly, no difference in antibiotic sensitivity of the clinical isolates tested in this study was observed when icariin was combined with the glycopeptide vancomycin. This observation is supported by work performed by Qin et al., who affirm that no synergism was observed when combining flavonoids with non-β-lactam antibiotics [9]. Interestingly, Liu et al. observed that the administration of icariin following antibiotic therapy with high-dose vancomycin for the resolution of bone infection decreased the adverse effects (delayed bone healing) related to high-dose vancomycin use [27]. This observation confirms evidence from previous studies where the osteoblastic properties of icariin are well described.

We present evidence to show that, in line with previously published studies, there does not seem to be a synergistic effect with the glycopeptide antibiotic vancomycin as with β-lactams AmoxyClav and ampicillin. There was no increase in sensitivity to vancomycin when co-incubated with icariin in the culture collection type strain, while the results in animal clinical isolates were variable and inconsistent.

Vital staining evidence confirmed that icariin enhanced AmoxyClav effectiveness resulting in greater antimicrobial activity than either compound on its own. When analyzing the CFU data, we demonstrate that the combination of icariin with AmoxyClav resulted in growth inhibition of 37% with respect to the antibiotic alone. Together, the data suggest that the combination has a greater power to inhibit growth.

For the first time, we describe the effect of icariin in combination with conventional antibiotics on clinical MRSA isolates from both humans and animals. We were able to demonstrate an icariin-specific, statistically significant increase in the proportion of dead cells and a corresponding decrease in viability as compared to control conditions. Similar effects of β-lactams in combination with other plant flavonoid extracts (epigallocatechin gallate [4,14,15,16,17,18,19,20] and epicatechin gallate catechin (EGCG) [9,10,19]) have been described previously. Hu et al. demonstrated an increase in antimicrobial sensitivity of β-lactamase-producing MRSA of between 8% and 32% when EGCG was co-incubated with ampicillin-sulbactan [19]. These values are similar to the increase in sensitivity that we observed in *mecA*+ animal clinical isolates in our experiment. Similarly, Qin et al. demonstrated that the combination of catechin together with epicatechin gallate increased the action of ampicillin, ampicillin-sulbactan, cefazolin, cefepime, and imipenen, all β-lactams commonly used in the treatment of infections caused by MRSA [9].

Previously published studies suggest that the activity of icariin-antibiotic combinations may be enhanced by introducing chemical substitutions and modifications in the flavonoid primary chemical structure [4]. Wang et al. demonstrated an increase in the antibacterial activity of icariin through the addition of aromatic sulfonyl groups. The antibacterial activity of icariin on its own against methicillin-resistant and sensitive strains of *S. aureus* was found to increase between 4% and 16% as compared to the reference activity of ciprofloxacin [6].

## 4. Materials and Methods

### 4.1. Icariin and Bacterial Strains

Purified icariin was purchased from Merck (catalog number: I1286-100MG). The standard culture collection *S. aureus* isolate was obtained from the Spanish Type Culture Collection (CECT 435). Human clinical isolates of MRSA were obtained from La Fe University Hospital in Valencia (Comunidad Valenciana, Spain) and Guadalajara University Hospital in Guadalajara (Castilla la Mancha, Spain). Animal clinical MRSA isolates were kindly provided by the PASAPTA-Pathology Group from CEU Cardenal Herrera University [31]. A list of all strains and sources is provided in Table 1.

### 4.2. Antibiograms

Antibiograms were performed using the Kirby-Bauer method, according to Clinical and Laboratory Standards Institute (CLSI) protocol M100 [32]. Amoxycillin-clavulanate, ampicillin, and vancomycin disks were obtained from Liofilchem (Italy) (Reference 9048, 9006, and 9045, respectively). Two different antibiograms were carried out in triplicate for every strain tested in this study; the first, a control antibiogram using antibiotics on its own according to CLSI protocols. For the second antibiogram, all test plates were pre-impregnated with 1% icariin (*w*/*v*). Briefly, 100 μL of a sterile 1% aqueous solution of icariin was spread on the surface of the plates and allowed to dry at room temperature in a sterile laminar flow cabinet. After this preliminary step, the CLSI protocol was followed exactly, beginning with the addition of the diluted bacterial culture and the addition of the antibiotic disks. All antibiograms were incubated at 37 °C for 18–24 h. Two independent investigators read each antibiogram (MCP and AMA), and disputes were resolved by a third investigator (MPO). The diameters of the inhibition zones were measured following incubation, and the mean value and standard deviation were calculated. A Student’s t-test was carried out to determine whether differences between test groups were statistically significant. A *p*-value < 0.05 was determined to denote a statistically significant difference.

### 4.3. Vital Staining

Vital staining with propidium iodide has been widely used previously to determine the degree of membrane disruption of *Staphylococcus aureus*, this being equated to cell viability [33]. An experiment was designed to determine the degree of membrane disruption caused by icariin in combination with the antibiotic panel. Triplicate cultures of the test strains were prepared, in which icariin (30 µg/mL) was co-incubated in conjunction with each antibiotic. Cultures were incubated at 37 °C with shaking at 110 rpm, and samples were taken at 3 and 6 h for analysis of viability using propidium iodide (Sigma-Aldrich, 79214) staining. Staining was carried out according to the manufacturer’s instructions. Briefly, a 1 mL sample of the suspension was taken at each time point and centrifuged at 13,000 rpm for 60 s in order to pellet the cells. The cells were then resuspended in phosphate-buffered saline (PBS). The cell pellet was washed twice more in PBS following the above protocol, after which 1 μL of PI solution (final concentration 1,5 μM) was added to a resuspended 1 mL microbial cell suspension and vortexed gently to mix. Microbial cells were incubated at room temperature for 5 min in the dark and immediately visualized using fluorescence/visible light microscopy in order to enumerate the dead cells (stained red) among the total cell count.

### 4.4. Calculation of Colony-Forming Units (CFU) to Determine Viability

The viability of icariin-treated cells post incubation was assessed by the standard CLSI method for determining the number of CFU. Triplicate cultures of the test strains were prepared, in which 30 µg/mL of icariin were co-incubated in conjunction with antibiotic AmoxyClav (1 µg/mL). Cultures were incubated at 37 °C with shaking at 110 rpm, and samples were taken at the 3 h time point for CFU analysis. Samples were diluted to an initial OD600 of 0.6, and 100 µL spread plated on brain-hear infusion (BHI) agar for 24 h, following which a colony count was performed. The results were compared with respect to the control.

## 5. Conclusions

This study is the first of its kind to demonstrate the enhancement effect of icariin in combination with key antibiotics used in the treatment of MRSA infections. A further strength of the study is the use of both human and animal clinical isolates, demonstrating the applicability of the research in multiple spheres as a strategy of one health. Our detection of the different behaviors of icariin in two common mutant backgrounds suggests a possible mechanism of action related to *mecA*+ isolates as compared to the more recently described *mecC*+ genotype. This type of observation may have important clinical and scientific ramifications and must be investigated further to decipher the mechanism of action of icariin and the reason for the differences in effectiveness in these two genetic backgrounds. Further research is also required to determine the optimum clinical concentration of icariin in combination with β-lactam antibiotics to allow the creation of a clinical protocol that, once tested in animal models, may be applied to human patients.

## Figures and Tables

**Table 1:** *S. aureus* strains and sources used in this study. CECT = Colección Española de Cultivos Tipo (Spanish Culture Type Collection).

Strain Name	Source
Control	*Staphylococcus aureus* Culture Collection Type Strain CECT 435
H-1	MRSA human isolate from La Fe University Hospital nº 24
H-2	MRSA human isolate from Guadalajara University Hospital nº 792943
H-3	MRSA human isolate from Guadalajara University Hospital nº 792945
H-4	MRSA human isolate from Guadalajara University Hospital nº 791426
H-5	MRSA human isolate from Guadalajara University Hospital nº 792765
H-6	MRSA human isolate from Guadalajara University Hospital nº 790623
H-7	MRSA human isolate from Guadalajara University Hospital nº 790473
H-8	MRSA human isolate from Guadalajara University Hospital nº 792362
H-9	MRSA human isolate from La Fe University Hospital nº 33
A-1	MRSA animal clinical isolate 1004 *mecA*+ genotype
A-2	MRSA animal clinical isolate 1032 *mecA*+ genotype
A-3	MRSA animal clinical isolate 990 *mecA*+ genotype
A-4	MRSA animal clinical isolate 987 *mecC*+ genotype
A-5	MRSA animal clinical isolate 999 *mecC*+ genotype

**Table 2:** CFU/mL of *S. aureus* after 3 h incubation in BHI liquid media (control) or BHI liquid media supplemented with icariin, AmoxyClav, or both.

CFU/mL	BHI	BHI + Icariin	BHI + AmoxyClav	BHI+ AmoxyClav+ Icariin
Average of 3 replicates	5.53 × 10^8^	5.94 × 10^8^	1.18 × 10^7^	8.61 × 10^6^
Standard deviation	3.87 × 10^8^	4.74 × 10^8^	7.77 × 10^6^	5.85 × 10^6^

## Data Availability

Data is contained within the article and supplementary material.

## Supplementary Materials

The following supporting information can be downloaded at: https://www.mdpi.com/article/10.3390/antibiotics11020233/s1, Table S1: Diameter inhibition zone of the antibiotics used in this study. All numbers represent the average values of at least three independent experiments.
