# Supplementary material for: Icariin in Combination with Amoxycillin-Clavulanate and Ampicillin, but Not Vancomycin, Increases Antibiotic Sensitivity and Growth Inhibition against Methicillin-Resistant Staphylococcus aureus
Source: Antibiotics (Basel). 2022 Feb 11;11(2):233. doi: 10.3390/antibiotics11020233 (PMC8868454; doi:10.3390/antibiotics11020233)
Supplement: Supplementary file 1 [file antibiotics-11-00233-s001.zip › antibiotics-1554950-supplementary.pdf]

Supplemetary Materials

Table S1. Diameter inhibition zone of the antibiotics used in this study. All numbers represent the average values of at least three independent experiments.

INHIBITION ZONE (MM)	AMOXYCLAV	AMPICILLIN	VANCOMYCIN	ICARIIN
**CONTROL STRAIN**	45,3	31,7	21,3	No inhibition
**H-1**	21	18,3	18,3	No inhibition
**H-2**	13,7	10,7	16,0	No inhibition
**H-3**	15,0	11,0	16,3	No inhibition
**H-4**	11,7	11,7	14,3	No inhibition
**H-5**	10,3	9,3	17,7	No inhibition
**H-6**	9,0	7,7	16,3	No inhibition
**H-7**	9,7	8,0	16,3	No inhibition
**H-8**	10,0	7,3	14,7	No inhibition
**H-9**	25,3	18,0	18,3	No inhibition
**A-1**	19,7	11,7	18,0	No inhibition
**A-2**	17,3	12,7	20,3	No inhibition
**A-3**	21,0	17,7	18,7	No inhibition
**A-4**	19,0	16,7	16,7	No inhibition
**A-5**	13,0	13,3	16,0	No inhibition
